# How Information on Superfoods Changes Consumers’ Attitudes: An Explorative Survey Study

**DOI:** 10.3390/foods11131863

**Published:** 2022-06-24

**Authors:** Bárbara Franco Lucas, Jorge Alberto Vieira Costa, Thomas A. Brunner

**Affiliations:** 1 Food Science and Management, School of Agricultural, Forest and Food Sciences (HAFL), Bern University of Applied Sciences, CH-3052 Zollikofen, Switzerland; barbarafranco_eng@hotmail.com; 2 Laboratory of Biochemical Engineering, College of Chemistry and Food Engineering, Federal University of Rio Grande (FURG), P.O. Box 474, Rio Grande 96203-900, RS, Brazil; jorgealbertovc@gmail.com

**Keywords:** consumer acceptance, attitude, attitude change, drivers

## Abstract

Increasing interest in healthy habits has created the market for what is commonly called “superfoods.” The goal of this study was to explore Swiss consumers’ initial and final attitudes toward superfoods as well as their change in attitude toward those foods after being provided selected information. A questionnaire survey was conducted to explore the individual traits of the respondents. The attitudes were assessed at the beginning and end of the survey. Four multiple regression analyses were performed. The results showed that consumers perceiving superfoods’ health benefits and expressing an interest in organic foods were associated with initial and positive attitudes. These predictors remained significantly related to the positive attitude at the end of the survey. Sociodemographic predictors (age and place of residence) were significant factors, with older people and individuals who lived in urban centers showing a higher propensity to improve their attitudes toward superfoods. Individuals with lower perceptions about the benefits of superfoods being healthy and lower levels of cultural participation showed a negative attitude change. Given that this study aims to shed light on the variables that influence the behavior of Swiss consumers toward the superfoods trend, it fills a significant gap in the literature.

## 1. Introduction

Nowadays, the demand for healthier and sustainable food, as well as social migration, local resources, and climate change, can cause changes in cultural identity and food behaviors [1,2]. These events are responsible for an increasing segment of consumers choosing balanced, healthy, environmentally friendly, and safe food [3,4].

Products with health claims include the so-called “superfoods”, a phenomenon increasingly explored [5,6,7,8]. The literature describes superfoods as a combination of functional properties with natural and exotic characteristics [8]. According to Shahbandeh [9], while the share of food and drink product launches using the term superfoods is high worldwide, it is especially pronounced in the United States and Germany. Tools such as Google Trends confirm the rising interest in superfoods. In addition, in recent years, several projects aiming to produce innovative superfoods (i.e., pomegranate and native herbs) have been launched [10,11]. 

Previous research reported that superfood consumption is higher among higher socioeconomic groups [7]. A recent segmentation study revealed six segments of consumers. Some of them showed positive attitudes toward superfoods while others presented skeptical/rejective attitudes [6].

Understanding the numerous reasons for buying and consuming foods is a complex process. According to Torri et al. [1], several factors, including availability, cost, nutritional value, and hedonic preferences, play an important role in food consumption. Characteristics such as health concerns and consumer knowledge could also play an important role. Identifying the preferences of consumers is essential to improving communication and marketing strategies [12]. Studies have explored consumer perceptions of functional foods [13,14] and superfoods [5,6,7,15,16], while others [17,18,19,20] have focused on nutritional properties of superfoods. However, to the best of our knowledge, none of these studies have explored consumers’ changing attitudes toward superfoods. In this context, the present study aims to explore the attitudes of respondents toward superfoods and measure changes in their attitudes after having read information about these foods.

## 2. Materials and Methods

### 2.1. Data Receiving and Sample

A questionnaire and a cover letter explaining the aim of the study were developed and sent randomly to selected Swiss residents by post. The Bern University of Applied Sciences approved the study and the survey was completed anonymously. Four hundred and seventy-nine questionnaires were returned (response rate of ~14%). After data cleaning where we removed questionnaires that presented more than half of missing responses or failed in the consistency test, 442 questionnaires remained and were used for the regression analyses [5]. The sample characteristics are displayed in Table 1.

### 2.2. Survey

The survey included questions designed to understand the respondents’ initial attitudes toward superfoods. Using a six-point numeric scale for the following four semantic differential scales previously used by Brunner et al. [21], the respondents were requested to state their attitudes toward superfoods: bad vs. good, unimportant vs. important, not to be supported vs. to be supported, and negative vs. positive. Internal consistency for this scale was measured using Cronbach’s α as 0.95. The abovementioned scale was used as a dependent variable in the first regression analysis to explore the respondents’ initial attitudes toward superfoods. 

Participants received a detailed description of superfoods according to Lucas et al. [5]. “Superfoods are recognized for the beneficial chemical composition with a high concentration of nutrients such as vitamins, minerals, and antioxidants that has possible health benefits. These foods are considered more than basic nutrition”. As examples, we cited some fruits, grains/seeds, leaves, and microalgae. This information was extracted from the literature [5,7,8,16,20]. The following questions of the survey measured the respondents’ adherence to validated constructs that we assumed are related to perceptions of superfoods. To reduce the length of the survey, the number of items on some of the scales was reduced. The scales used in this study are shown in Table 2. 

Five sociodemographic questions were asked at the end of the survey. Finally, the respondents’ attitudes toward superfoods were evaluated once again using the scale presented previously. This construct represented the dependent variable for the second regression analysis.

### 2.3. Data Analyses

IBM SPSS Statistics (version 25) was used to analyze the data. The reliability of the scales was accessed using Cronbach’s α. Multiple linear regression analyses using the backward method with removal criteria higher than 0.05 were conducted to explore the influence of the 29 predictors (independent variables) on the dependent variables (first regression: attitude at the beginning of the survey; second regression: attitude at the end of the survey). The collinearity diagnostics did not reveal any concerns about the regression analyses [22]. To explore the two dependent variables of attitude change, the attitude expressed at the beginning of the survey was subtracted from the attitude conveyed at its end.

**Table 2:** Scales used in the questionnaire and their reliability scores.

Scale	Example of an Item	Number of Items	Range	Cronbach’s *α*	Reference
Previous knowledge	I’ve already read/heard a lot about superfoods	4	1 (strongly disagree) to 6 (strongly agree)	0.91	[21,23]
Health benefit perception	Superfoods offer a significant health benefit	3	1 (strongly disagree) to 6 (strongly agree)	0.92	Based on [21]
Sustainable benefit perception	Superfoods offer a significant advantage in terms of sustainability	3	1 (strongly disagree) to 6 (strongly agree)	0.92	Based on [21]
Cooking creativity	When I cook, I like to try new recipes.	3	1 (strongly disagree) to 6 (strongly agree)	0.86	[21]
Subject vitality	I feel alive and vital.	7	1 (not at all true) to 6 (very true)	0.86	[24]
Self-reported health status	I feel physically healthy.	3	1 (strongly disagree) to 6 (strongly agree)	0.80	[25]
Cultural participation	How often do you visit: an art museum?	5	1 (never) to 6 (very often)	0.82	[7]
Food neophobia	I am afraid to eat things I have never had before.	10	1 (strongly disagree) to 6 (strongly agree)	0.81	[26]
Food technology neophobia	There is no sense trying out high-tech food products because the ones I eat are already good enough.	4	1 (strongly disagree) to 6 (strongly agree)	0.83	[27]
General health interest	I am very particular about the healthiness of food I eat.	3	1 (strongly disagree) to 6 (strongly agree)	0.66	[28]
Price–quality relation	I always try to get the best quality for the best price.	3	1 (strongly disagree) to 6 (strongly agree)	0.62	[29]
Food involvement	I enjoy cooking for others and myself.	4	1 (strongly disagree) to 6 (strongly agree)	0.64	[30]
Environmental protection ^1^	Has been prepared in an environmentally friendly way.	3	1 (strongly disagree) to 6 (strongly agree)	0.92	[31]
Satisfaction with food-related life	My life in relation to food and meals is close to my ideal.	5	1 (strongly disagree) to 6 (strongly agree)	0.71	[32]
Safety ^2^	Whether I am certain it does not contain harmful bacteria or viruses.	3	1 (not at all) to 5 (very)	0.62	[33]
Convenience ^2^	How easy or difficult it is to prepare.	3	1 (not at all) to 5 (very)	0.79	[33]
Health/weight concern ^2^	How likely it is to help me control my weight.	3	1 (not at all) to 5 (very)	0.87	[33]
Comfort ^2^	How much it will help me relax.	3	1 (not at all) to 5 (very)	0.80	[33]
Sensory appeal ^2^	How it tastes.	3	1 (not at all) to 5 (very)	0.52	[33]
Organic ^2^	Degree to which it contains natural ingredients.	4	1 (not at all) to 5 (very)	0.78	[33]
Accessibility ^2^	Whether it can be bought in shops close to where I live or work.	3	1 (not at all) to 5 (very)	0.48	[33]
Tradition ^2^	Degree to which it reflects my cultural or ethnic traditions.	3	1 (not at all) to 5 (very)	0.70	[33]
Nutritional knowledge ^3^	Fat is always bad for your health; you should therefore avoid it as much as possible ^R^.	6	0 (false), 1 (I don’t know), 2 (true) (maximum sum of scores = 12)	0.52	[34]

^1^ Introductory statement: Is important to me that the food I eat on a typical day. ^2^ Introductory statement: When deciding what foods to buy or eat on a daily basis, how important are each of the following? ^3^ Introductory statement: Please indicate, in your opinion, if these statements are true or false. ^R^ Reversed for analyses.

## 3. Results

### 3.1. Respondents’ Initial Attitudes toward Superfoods

The initial attitudes of the respondents toward superfoods were somewhat positive, with M = 3.89 (SD = 1.32). A multiple regression analysis was performed considering the initial attitude as the dependent variable, and the 29 constructs related to the behavioral patterns and sociodemographic variables were considered as the predictors. Five of the constructs showed a significant contribution to the model (Model 1), explaining 55% of the variance (Table 3). 

Health benefit perception showed the strongest influence, followed by previous knowledge, and both were related to a more positive attitude toward superfoods. 

The importance of organic nature was also positively related to the initial attitude. On the contrary, the predictors food technology neophobia and environmental protection appeared to be related to a more negative attitude toward superfoods. The lower the phobia toward novel technologies producing food and the lower the interest in protecting the environment, the more favorable the attitude toward superfoods. 

### 3.2. Respondents’ Final Attitudes toward Superfoods

The final attitudes of the respondents toward superfoods were also positive, with M = 4.21 (SD = 1.30). Health benefit perception, food technology neophobia, organic, and previous knowledge remained relevant predictors after the respondents were provided with the information (Table 4); however, previous knowledge lost some of its predictive power. 

Sustainability benefit perception emerged as a positively related determinant of attitude. The interest in environmental protection, which was previously significant and inversely related to attitude, disappeared at the end of the survey.

Convenience and sensory appeal emerged as weak, yet nonetheless significant, predictors of a positive final attitude toward superfoods. The higher the interest of the respondents toward food pleasing the senses and easy-to-prepare foods, the more favorable their attitudes toward superfoods. Tradition appeared to be a negative predictor: the less the respondents cared about following their traditions when eating or buying food, the more positive their attitudes toward superfoods. This model (Model 2) explained 61% of the variance.

### 3.3. Predicting Attitude Change among the Respondents

Two additional multiple regressions were conducted to predict the changes in attitudes while filling in the questionnaire and gaining information about the topic. The variables that were significantly related to positive and negative attitude changes are displayed in Table 5. In 220 cases, the attitude improved, and the resulting positive difference was used as the dependent variable for the third regression. In 99 cases, the attitude changed negatively, and the moduli of the differences were used for the fourth regression analysis. Participants with no change in attitude (*n* = 103) were not considered in these models of attitude change.

The lower the respondents’ previous knowledge and interest in the accessible food products, the higher the extent of their positive attitude changes. The importance of an acceptable price–quality relationship and vitality also resulted in more pronounced positive attitude changes. In addition, older people and those living in urban centers showed greater opinion improvements.

A negative attitude change was observed in participants with a lower perception of superfoods being healthy. Concerning cultural participation, the less the respondents reported being involved in such activities, the greater the degree of their negative attitude changes. Lower food technology neophobia was related to a more negatively pronounced change. In addition, the negative attitude change was higher among safety-oriented consumers. Age, previous knowledge, and the price–quality relationship influenced attitudes in an opposing way compared to the model for the positive attitude change (Model 3). The older the respondents, the lower their extent of previous knowledge, and the greater the importance they accorded to the price–quality relationship, the less negative their changes.

## 4. Discussion

The present work resulted in an understanding of the variables that can predict the attitudes and attitude changes of Swiss consumers toward superfoods. The information in the survey significantly impacted the respondents’ opinions, as shown in the second rating of their attitudes. A detailed discussion of the findings is presented below.

### 4.1. Drivers of Initial Attitudes toward “Superfoods”

According to our results, the respondents who initially showed a positive attitude toward superfoods also reported a higher health benefit perception as well as a high interest in the organic nature of these foods. Previous research showed that consumers associate organic products with healthier nutrition [35]. 

Respondents with a more positive attitude also showed lower phobia toward new technologies used to produce foods. Similarly, Caracciolo et al. [36] reported a low neophobia toward food technology associated with the consumption of dietary supplements in Italy.

A negative effect of the variable environmental protection on attitudes toward superfoods was observed. Thus, the more consumers care about environmental protection, the lower they score on attitude toward superfoods. These scores were obtained before we presented the sustainability-related arguments in favor of superfoods, and they are probably related to the fact that some respondents, at that point, assumed that most superfoods originate in distant countries, resulting in emissions connected to their transport [37]. 

### 4.2. Drivers of Final Attitudes toward “Superfoods”

Additional information on a specific claim can increase the value perceived by the consumer [2]. In the present study, three positive predictors (health benefit perception, previous knowledge, and organic), which were found to be relevant at the beginning of the survey, remained significant at the end of the survey. Moreover, the negative predictor of food technology neophobia persisted as a significant factor. Another important finding is that the information and the sustainability-related arguments provided during the survey helped the respondents to overcome their concerns about environmental protection, i.e., a predictor that was not significant in the second regression.

Furthermore, new significant predictors appeared: sustainability benefit perception, tradition, convenience, and sensory appeal. The positive and significant influence of the variable sustainability benefit perception illustrated that we provided strong arguments about superfoods being sustainable.

The findings of this work indicated that the lower the level of interest in eating or buying familiar, recognizable, and traditional foods, the higher the attitude scores in favor of superfoods. According to van den Driessche et al. [38], some superfoods have only been recently introduced into the Western diet. This finding is in agreement with our results. Therefore, superfoods are not considered part of traditional meals for Swiss citizens.

The significance of convenience revealed that consumers interested in easy and quick food preparation showed a positive attitude toward superfoods at the end of the survey. Additionally, consumers interested in foods that are pleasing to their senses (e.g., in terms of taste and appearance) scored high in attitude. Previous research reported that sensory appeal is also predictive of the attitude toward foods with health claims [39]. According to the authors, their participants not only perceived functional foods as healthy, but they also expected to eat tasty and sensorily pleasant food.

### 4.3. Drivers for Attitude Change

Most consumers changed their attitudes, eventually favoring superfoods. Thus, our survey succeeded in increasing the positive perception of these foods. Pre-existing general knowledge about superfoods was a positive predictor of attitude in both regressions (Table 3 and Table 4); however, when we evaluated the attitude change, previous knowledge appeared to be a negative predictor and acted as a barrier hindering positive attitude change. Based on these results, the less the respondents knew, the more they could learn during the survey, and the more positive their changes in attitude were. Brunner et al. [21] observed that consumers who affirm to have pre-existing knowledge about 3D food printing retained their impressions even after receiving the new information.

Lyerly and Reeve [33] defined accessibility as “the degree to which food is easy to access physically (e.g., available at local stores) and financially (e.g., cost).” In the present study, respondents with low interest in accessibility showed higher increases in their attitude scores. This result suggests that consumers do not care about a small level of inconvenience when buying superfoods. This finding is supported by the results of Lucas et al. [5]. According to Dang et al. [40], the use of online food services by consumers has been rising in recent years. Results on the price–quality relationship demonstrated that the respondents who changed their views on superfoods to a more positive one by the end of the survey also desired foods of good quality and compared food prices when shopping.

Although the sociodemographic predictors did not show significant results in the first two regression models, age and place of residence were significant in the model that predicted attitude change. Older people and those living in urban areas expressed fewer reservations against superfoods and showed a positive attitude change. Similarly, a previous study reported the effect of age on the acceptability of “exotic” food [1]. It was also noted that the area of residence is an important factor affecting the consumption attitude regarding exotic foods [1].

According to Ryan and Frederick [24], vitality is related to feelings of aliveness and energy. In the present research, respondents who claimed to experience these feelings showed a greater propensity to change their attitudes positively. It is well known that “health claims” are the most powerful claims surrounding the superfoods trend and that this argument differentiates these foods from their conventional counterparts. Therefore, the respondents who expressed reservations about the health benefits of superfoods also showed a negative attitude change concerning them.

The less the phobia about the novel technologies used to produce food, the more negative the attitude change. This result might be attributed to our presentation of the information on superfoods as a natural (not highly processed) product, and at the end of the survey, the respondents who were less afraid of new technologies were perhaps disappointed, because they might want to consume superfoods that are highly processed.

Surprisingly, the change in a negative attitude was significantly higher among respondents with a higher interest in safety. This may be attributed to the fact that most of the exotic superfoods named in the information originate in countries located far away and that are typically less developed compared to Switzerland. These aspects might have led to safety concerns among respondents with this particular mind-bending point.

In the present study, the lower the respondents’ participation in cultural activities, the higher the extent of negative change in the attitude toward superfoods. This result is following the finding reported by Groeniger et al. [7], which reported that cultural participation is associated with superfood consumption. 

### 4.4. Limitations and Future Research

As the term superfoods is not yet regulated in Switzerland and worldwide, some of the people to whom the survey was sent may have been confused, leading them to avoid answering the questionnaire and resulting in a low response rate (below 15%) [5,6]. Future research may address this issue by providing a more interactive approach (e.g., online questionnaires), including pictures of superfoods. In Switzerland, superfoods received some media attention and some retailers included the concept in their marketing activities. Nevertheless, we think that most consumers are neither involved in superfoods nor know much about it in depth. The information in the questionnaire might have been the first real confrontation with the topic for most respondents. Therefore, we argue that a real change in attitude might have occurred for these consumers since they learned about the concept throughout the questionnaire. This observed change in attitude might also be rather stable. For other respondents with previous knowledge, the questionnaire might have only increased the salience, which in turn might have affected the attitude in the end. In this case, this change might not be very stable. More research is needed to investigate attitude change over time. 

Our study was explorative by nature. Our goal was to focus on potentially directly related variables and to identify the drivers and barriers to superfood consumption. The next step could focus on putting the identified variables into a broader model such as the Theory of Planned Behavior. In such a study, subjective norms and perceived behavioral control should be incorporated.

## 5. Conclusions

The present study attempted to explore the factors that influence Swiss consumers’ attitudes toward superfoods. The initial and final attitudes, as well as the attitude changes, were analyzed in three steps. Three strong positive predictors (health benefit perception, previous knowledge of superfoods, and organic) were found to be related to a positive attitude toward superfoods at the beginning and end of the survey.

The well-designed information on superfoods provided in the survey resulted in a shift in attitude for most respondents (52%). Concerns about environmental protection were overcome, and sustainability benefit perception was found to be a significant and positive predictor at the end of the survey. The drivers identified and analyzed in this study could be used by superfoods researchers, producers, and marketers to develop new superfood products and communication strategies.

## Figures and Tables

**Table 1:** Characteristics of the studied sample (*n* = 422).

Parameters	Sample (%)	Parameters	Sample (%)
Sex		Education level	
Female	55	None	1
Male	45	Compulsory school	2
Residence place		Apprenticeship	33
Urban	51	Secondary/high School	8
Rural	49	Higher technical and vocational training	20
Age groups		University of Applied Sciences	18
18–35 years	14	University	19
36–50 years	27		
51–65 years	33	Household size	
66–79 years	20	1 person	20
80 years or more	7	2 persons	40
		3 persons	12
Occupation		4 persons	21
Full/part time	64	5 persons	6
Not working	36	6 persons or more	1

**Table 3:** Results from multiple linear regression analyses explaining consumers’ initial attitudes toward superfoods.

Variable	B	SE (B)	β	*p*
Constant	1.81	0.32		0.000
Health benefit perception	0.53	0.04	0.57	0.000 ***
Previous knowledge	0.17	0.03	0.20	0.000 ***
Food technology neophobia	−0.22	0.04	−0.19	0.000 ***
Organic	0.27	0.09	0.15	0.003 **
Environmental protection	−0.13	0.06	−0.10	0.033 *

Note. *R*^2^ = 0.55. ***, **, and * denote *p* < 0.001, <0.01, and 0.05, respectively. *n* = 422.

**Table 4:** Results from multiple linear regression analyses explaining respondents’ final attitudes toward superfoods.

Variable	B	SE (B)	β	*p*
Constant	1.67	0.37		0.000
Health benefit perception	0.32	0.05	0.36	0.000 ***
Sustainability benefit perception	0.24	0.05	0.26	0.000 ***
Food technology neophobia	−0.25	0.04	−0.22	0.000 ***
Tradition	−0.16	0.05	−0.12	0.001 **
Organic	0.18	0.06	0.10	0.004 **
Previous knowledge	0.08	0.03	0.10	0.004 **
Convenience	0.12	0.05	0.08	0.015 *
Sensory appeal	0.14	0.07	0.07	0.034 *

Note. *R*^2^ = 0.61. ***, **, and * denote *p* < 0.001, <0.01, and 0.05, respectively. *n* = 422.

**Table 5:** Results from multiple linear regression analyses explaining respondents’ attitude changes toward superfoods.

Attitude Change	Variable	B	SE (B)	β	*p*
Positive (Model 3)	Constant	0.65	0.42		0.125
	Previous knowledge	−0.11	0.04	−0.20	0.004 **
	Accessibility	−0.18	0.08	−0.16	0.023 *
	Price–quality relation	0.11	0.05	0.14	0.049 *
	Age	0.01	0.00	0.14	0.033 *
	Place of residence	0.22	0.10	0.14	0.038 *
	Vitality	0.12	0.06	0.14	0.043 *
Negative (Model 4)	Constant	1.60	0.62		0.012
	Health benefit perception	−0.17	0.05	−0.36	0.002 **
	Food technology neophobia	−0.18	0.07	−0.31	0.006 **
	Age	−0.02	0.01	−0.31	0.003 **
	Previous knowledge	0.13	0.05	0.30	0.006 **
	Price–quality relation	−0.25	0.10	−0.27	0.012 **
	Safety	0.19	0.09	0.24	0.029 **
	Cultural participation	−0.14	0.07	−0.22	0.045 **

Note: ** and * denote <0.01 and 0.05, respectively. Coding for place of residence: 0 = rural, 1 = urban. For a positive attitude change, *R*^2^ = 0.114 and *n* = 220. For a negative attitude change, *R*^2^ = 0.272 and *n* = 99.

## Data Availability

Data are available upon reasonable request.
